# Deep learning for atrioventricular regurgitation diagnosis: an external validation study

**DOI:** 10.1093/ehjdh/ztaf078

**Published:** 2025-07-15

**Authors:** Ido Cohen, Jeffrey G Malins, Michal Cohen-Shelly, Yossi Asaf, Michael Fiman, Kobi Faierstein, Lior Fisher, Karin Sudri, Ehud Raanani, Ehud Schwammenthal, Robert Klempfner, Elad Maor

**Affiliations:** Leviev Cardiovascular Institute, Sheba Medical Center, Derech Sheba 2, Tel HaShomer, Ramat Gan 52621, Israel; Gray Faculty of Medical and Health Sciences, Tel Aviv University, Ramat Aviv, Tel Aviv 69978, Israel; Department of Cardiovascular Medicine, Mayo Clinic, 200 First Street SW, Rochester, MN 55905, USA; Leviev Cardiovascular Institute, Sheba Medical Center, Derech Sheba 2, Tel HaShomer, Ramat Gan 52621, Israel; Aisap.ai, Derech Sheba 2, Ramat Gan 5266202, Israel; ARC Innovation Center, Sagol AI Hub, Sheba Medical Center, Derech Sheba 2, Ramat Gan 52621, Israel; Leviev Cardiovascular Institute, Sheba Medical Center, Derech Sheba 2, Tel HaShomer, Ramat Gan 52621, Israel; Gray Faculty of Medical and Health Sciences, Tel Aviv University, Ramat Aviv, Tel Aviv 69978, Israel; Aisap.ai, Derech Sheba 2, Ramat Gan 5266202, Israel; Leviev Cardiovascular Institute, Sheba Medical Center, Derech Sheba 2, Tel HaShomer, Ramat Gan 52621, Israel; Gray Faculty of Medical and Health Sciences, Tel Aviv University, Ramat Aviv, Tel Aviv 69978, Israel; Leviev Cardiovascular Institute, Sheba Medical Center, Derech Sheba 2, Tel HaShomer, Ramat Gan 52621, Israel; Gray Faculty of Medical and Health Sciences, Tel Aviv University, Ramat Aviv, Tel Aviv 69978, Israel; ARC Innovation Center, Sagol AI Hub, Sheba Medical Center, Derech Sheba 2, Ramat Gan 52621, Israel; Leviev Cardiovascular Institute, Sheba Medical Center, Derech Sheba 2, Tel HaShomer, Ramat Gan 52621, Israel; Gray Faculty of Medical and Health Sciences, Tel Aviv University, Ramat Aviv, Tel Aviv 69978, Israel; Aisap.ai, Derech Sheba 2, Ramat Gan 5266202, Israel; Leviev Cardiovascular Institute, Sheba Medical Center, Derech Sheba 2, Tel HaShomer, Ramat Gan 52621, Israel; Gray Faculty of Medical and Health Sciences, Tel Aviv University, Ramat Aviv, Tel Aviv 69978, Israel; Aisap.ai, Derech Sheba 2, Ramat Gan 5266202, Israel; Leviev Cardiovascular Institute, Sheba Medical Center, Derech Sheba 2, Tel HaShomer, Ramat Gan 52621, Israel; Gray Faculty of Medical and Health Sciences, Tel Aviv University, Ramat Aviv, Tel Aviv 69978, Israel; Aisap.ai, Derech Sheba 2, Ramat Gan 5266202, Israel; Leviev Cardiovascular Institute, Sheba Medical Center, Derech Sheba 2, Tel HaShomer, Ramat Gan 52621, Israel; Gray Faculty of Medical and Health Sciences, Tel Aviv University, Ramat Aviv, Tel Aviv 69978, Israel; Aisap.ai, Derech Sheba 2, Ramat Gan 5266202, Israel

**Keywords:** Artificial intelligence, Machine learning, External validation, Echocardiography, Mitral regurgitation, Tricuspid regurgitation, Atrioventricular valve regurgitation

## Abstract

**Aims:**

Mitral and tricuspid regurgitation (MR and TR) are common in older adults and associated with substantial morbidity and mortality. While transthoracic echocardiography (TTE) is the diagnostic gold standard, access remains limited in many care settings. Artificial intelligence (AI)–based echocardiographic analysis may help address this diagnostic gap.

**Methods and results:**

We externally validated a deep learning algorithm developed by Aisap.ai using TTE studies from the Mayo Clinic Health System (2013–23). The model analyses echocardiographic images to classify atrioventricular regurgitation severity and was evaluated against cardiologist interpretations. Performance was assessed using binary (normal–mild vs. moderate–severe) and ordinal (normal, mild, moderate, severe) classification schemes. Among 1541 eligible TTEs, the model returned predictions for 578 studies (38%). Performance analysis was limited to these cases. The MR cohort included 280 studies and the TR cohort 298. For MR, the model achieved an area under the receiver operating characteristic curve (AUC) of 0.98 [95% confidence interval (CI): 0.97–0.99], with 91% accuracy, 95% sensitivity, and 89% specificity. For TR, the AUC was 0.96 (95% CI: 0.94–0.98), with 84% accuracy, 91% sensitivity, and 80% specificity.

**Conclusion:**

In cases where a prediction was generated, the model demonstrated high diagnostic performance in identifying clinically significant atrioventricular regurgitation. These findings support the feasibility of AI-assisted echocardiography in diverse populations, while underscoring the need for technical alignment between model requirements and local acquisition practices to ensure real-world applicability.

## Graphical Abstract

**Graphical Abstract:**
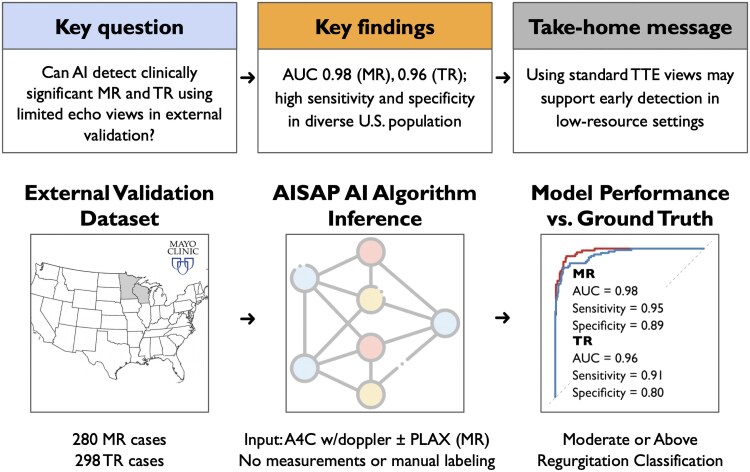
External validation of a deep learning algorithm (Aisap.ai) for detecting moderate-or-greater mitral and tricuspid regurgitation using limited transthoracic echocardiography views. The model was tested on 578 studies from the Mayo Clinic (280 mitral regurgitation, 298 tricuspid regurgitation), analysing A4C ± PLAX views with colour Doppler. Diagnostic performance was high (mitral regurgitation: area under the receiver operating characteristic curve 0.98, sensitivity 0.95, specificity 0.89; tricuspid regurgitation: area under the receiver operating characteristic curve 0.96, sensitivity 0.91, specificity 0.80). Findings suggest potential for artificial intelligence–assisted triage in non-specialist or resource-limited settings.

## Introduction

Mitral and tricuspid regurgitation (MR and TR) are common valvular heart diseases, particularly in adults over the age of 65, among whom moderate to severe MR affects an estimated 2.3–6.9% and TR 2.7–7.2%.^1,2^ Both conditions are associated with substantial morbidity, including increased rates of heart failure hospitalization and all-cause mortality.^3–7^ Treatment options have expanded in recent years to include not only surgical and medical management but also a growing range of transcatheter interventions, such as edge-to-edge repair and valve replacement.^8–11^ These advances underscore the clinical importance of timely and accurate diagnosis.

In routine clinical practice, the evaluation of suspected valvular disease typically relies on history taking and physical examination. History is often uninformative, as MR and TR may be asymptomatic or present with symptoms that overlap with various cardiac and non-cardiac conditions, such as dyspnoea or fatigue. Physical examination remains a crucial component of bedside assessment, but its sensitivity and specificity for detecting MR and TR are modest, and grading severity can be particularly challenging in non-specialist settings.^12^ Consequently, clinically significant regurgitation may go unrecognized, while positive but non-specific findings, such as soft systolic murmurs or functional flow-related sounds, can introduce uncertainty regarding which patients warrant formal echocardiographic evaluation.

In contrast, comprehensive transthoracic echocardiography (TTE) serves as the diagnostic gold standard for assessing valvular regurgitation. Accurate interpretation requires high-quality image acquisition across standardized views, Doppler interrogation, and quantitative assessment of parameters such as effective regurgitant orifice area, regurgitant volume, and pulmonary vein flow.^13–15^ This process is technically demanding, relying on trained sonographers for acquisition and experienced cardiologists for integrated interpretation. Even with optimal technique, inconsistencies among parameters,^16,17^ interobserver variability,^18–20^ and the dynamic nature of regurgitation—affected by preload, afterload, rate and rhythm^21–24^—can complicate severity assessment. These limitations, combined with the resource-intensive nature of formal TTE, constrain its scalability, particularly in primary and decentralized care settings.

There is therefore an unmet need for accessible, scalable tools that support early identification of clinically relevant valvular disease in primary care and out-of-hospital environments. Artificial intelligence (AI)–assisted echocardiography^25,26^ offers a promising approach to bridge this diagnostic gap. One potential application is the use of AI algorithms to assist non-expert users, particularly in conjunction with point-of-care ultrasound (POCUS), by automating image analysis and interpretation. This may facilitate earlier detection in settings where formal echocardiography is unavailable or delayed.

Aisap.ai is a Food and Drug Administration (FDA)–cleared AI solution designed to support the echocardiographic diagnosis of structural heart disease, including MR and TR.^27,28^ Its intended use is to augment frontline clinicians, especially in primary care, urgent care, skilled nursing facilities, and rural hospitals, by providing real-time, automated interpretation of echocardiographic loops. In doing so, it addresses the need for more equitable access to cardiac imaging and timely referral of patients who may benefit from pharmacological or interventional therapy.

In this study, we retrospectively evaluated the performance of the Aisap.ai algorithm in diagnosing atrioventricular valve regurgitation using formal TTEs acquired across community-based satellite sites within the Mayo Clinic Health System (MCHS) across Minnesota and Wisconsin. This external validation aimed to assess the algorithm’s generalizability across a diverse US population and varied clinical workflows, reflecting environments where the model is intended to be deployed.

## Methods

### Study design and cohort selection

**Figure 1:**
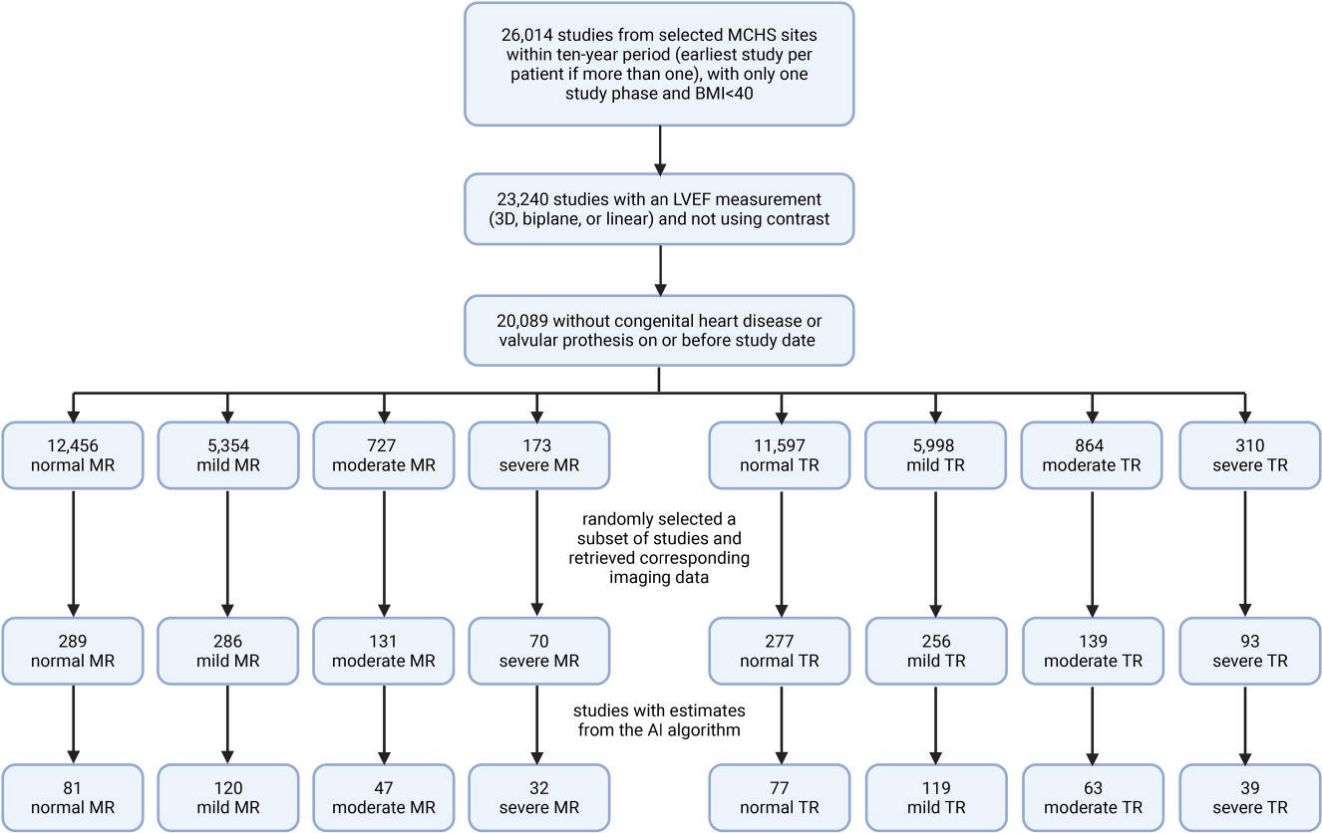
Cohort selection flow chart.

This retrospective external validation study assessed the diagnostic performance of the Aisap.ai algorithm in evaluating mitral and tricuspid regurgitation. The dataset (*Figure 1*) comprised TTEs performed between September 2013 and September 2023 at selected satellite sites of the MCHS located in Minnesota and Wisconsin.

Inclusion criteria encompassed resting, single-phase (baseline) TTEs from patients aged over 21 years with a body mass index below 40 kg/m². Examinations were excluded if they involved contrast agents, did not have a measurement for left ventricular ejection fraction (3D volume, biplane, or linear), or had ICD codes for congenital heart disease or valvular prosthesis noted in the patient’s medical record on or before the echocardiographic study date. For patients with multiple eligible exams within the date range, the earliest exam was selected.

From the full set of candidate exams, studies were identified with ground truth labels (as specified in the section Ground truth labelling) corresponding to one of the following degrees of regurgitation for MR or TR: normal (either ‘no’ or ‘trivial’), mild, moderate, or severe. Then, a random sample of studies for each of the severity levels of MR and TR was identified using the *sample* function in version 1.5.0 of the *pandas* library in Python version 3.9.13, and corresponding DICOM (Digital imaging and communications in medicine) data were retrieved (if available) from Notion, an internal Mayo Clinic platform for accessing echocardiographic imaging. These exams were processed by the model and corresponding AI results were obtained for a fraction of these exams, as shown in the bottom row of *Figure 1*.

All study procedures received approval from the Mayo Clinic Institutional Review Board and included only patients who provided consent for the use of their data in research.

### Aisap.ai algorithms overview

Aisap.ai is a deep learning–based software platform comprising two integrated modules: a view classification and quality assurance model and a diagnostic model trained to assess cardiac conditions based on limited echocardiographic inputs.

The view classification model identifies the echocardiographic view type and assesses input quality assuring that the input is suited for the diagnostic model. It assigns each video loop to one of 18 standard transthoracic echocardiographic views using frame-wise classification and a probability-based voting mechanism. The model was trained on 12 000 loops annotated by sonographers. Loops are accepted only if they contain sufficient consecutive frames exceeding a predefined confidence threshold. In parallel, the model computes a continuous image quality score ranging from 0 to 1, automatically excluding loops that fall below a defined quality threshold. This ensures that only diagnostically adequate inputs are passed to the diagnostic model.

The diagnostic model was developed and internally validated using over 140 000 TTE studies acquired at Sheba Medical Center between 2007 and 2021. All studies adhered to American Society of Echocardiography (ASE) standards and were labelled using final clinical interpretations by board-certified cardiologists. The model was trained via supervised learning to assess severity classifications assigned by clinicians.

Importantly, the model does not rely on guideline-based measurements such as proximal isovelocity surface area (PISA), vena contracta width, or effective regurgitant orifice area (EROA); instead, it infers severity based on learned visual patterns. At inference, Aisap.ai assigns a probability to each severity class (normal, mild, moderate, severe) and selects the most likely category. Following internal validation, the model was locked and remained unchanged during external testing; no retraining or tuning was performed on the external cohort.

For MR, Aisap.ai processes parasternal long-axis (PLAX) and apical four-chamber (A4C) views with colour Doppler. For TR, it uses the A4C view with colour Doppler alone. From each video loop, 10 frames spanning a 1.5-s interval are automatically selected to ensure temporal coverage of the cardiac cycle. For MR assessment, PLAX and A4C inputs are merged into a unified model input structure.

The model architecture combines convolutional neural networks (CNNs) with transformer-based attention mechanisms. Feature vectors are extracted from each sampled frame using EfficientNetV2^29^ and then passed through multi-head self-attention transformer^30^ blocks to capture temporal relationships. These outputs are max-pooled into a single 1024-dimensional vector, which is processed by a classification head to predict regurgitation severity. This architecture enables integration of both spatial and temporal features from 2D and colour Doppler imaging.

### Ground truth labelling

All echocardiographic studies were conducted as part of routine clinical care at MCHS sites using commercially available ultrasound systems. Image acquisition followed institutional protocols aligned with ASE guidelines and included all standard views, Doppler modalities, and quantitative measurements necessary for comprehensive valvular assessment. Studies were acquired by credentialed sonographers and interpreted by staff echocardiography physicians with level III training.^31^

Ground truth labels for mitral and tricuspid regurgitation severity were derived from the final clinical interpretations documented in formal echocardiographic reports and were extracted using SQL queries from an internal IBM DB2 database, which was transitioned to Google Cloud Platform (GCP) BigQuery later in the study period. These assessments were performed independently, without any access to or influence from the Aisap.ai model or its outputs.

### Model inference and output generation

DICOM files were uploaded to a secure GCP virtual machine and processed using version 1.0 of the model via a command-line interface, with AI-generated results returned on the order of minutes per study.

For each echocardiogram in the cohort, the model produced an automated severity classification prediction, which was paired with the corresponding ground truth label from the clinical report. Notably, the model was applied retrospectively in a fully automated, back-end fashion—outside of its intended real-time, user-facing workflow—and without any human interaction or feedback. No outputs were visible to clinicians or incorporated into clinical care during the study period.

### Statistical analysis

For each of the two target pathologies—mitral and tricuspid regurgitation—model performance was evaluated using three main approaches. First, the algorithm’s ability to distinguish clinically significant regurgitation (moderate or severe) from non-significant regurgitation (normal or mild) was assessed using standard binary classification metrics: area under the receiver operating characteristic curve (AUC), sensitivity, specificity, accuracy, and F1 score.

Second, a one-vs-rest analysis was conducted for each individual severity class (normal, mild, moderate, severe) using the same metrics to evaluate class-specific performance.

Third, agreement between Aisap.ai predictions and ground truth labels was visualized using 4 × 4 confusion matrices reflecting the multiclass classification results.

Baseline characteristics of the study cohorts (*Table 1*) were summarized using median and interquartile range (IQR) for continuous variables and absolute counts with percentages for categorical variables. Comorbidities were ascertained using the comorbidity package in R, based on ICD-9 or ICD-10 codes documented in the patient’s medical record up to and including the date of the echocardiographic study.^32^ All analyses were conducted using R software (version 4.3.3, R Foundation for Statistical Computing).

**Table 1:** Mitral regurgitation and tricuspid regurgitation cohort baseline clinical and echocardiographic characteristics

Characteristics	MR (*n* = 280)	TR (*n* = 298)
Age	76 [66–82]	76 [66–84]
Male	126 (45)	151 (50.7)
Race		
Non-Hispanic white	266 (95)	287 (96.3)
Hispanic/Latino	4 (1.4)	4 (1.3)
Asian	3 (1.1)	1 (0.3)
Black	1 (0.4)	1 (0.3)
Other	6 (2.1)	5 (1.7)
Coronary artery disease	54 (19.3)	70 (23.5)
Arrhythmia	202 (72.1)	234 (78.5)
LVEF (%)	57 [51–63]	56 [48–62]
Cerebrovascular disease	97 (34.6)	108 (36.2)
Dementia	22 (7.9)	20 (6.7)
Peripheral vascular disease	114 (40.7)	126 (42.3)
Chronic pulmonary disease	113 (40.4)	115 (38.6)
Pulmonary circulation disorder	46 (16.4)	51 (17.1)
Renal disease	108 (38.6)	134 (45)
Liver disease	8 (2.9)	10 (3.4)
Diabetes	57 (20.4)	64 (21.5)
Hypertension	165 (58.9)	193 (64.8)
Regurgitation ground truth		
Normal	81 (28.9)	77 (25.8)
Mild	120 (42.9)	119 (39.9)
Moderate	47 (16.8)	63 (21.1)
Severe	32 (11.4)	39 (13.1)
Manufacturer		
GE	257 (91.8)	256 (85.9)
Philips	23 (8.2)	42 (14.1)

Values are *n* (%) for categorical variables or median [IQR] for continuous variables.

MR, mitral regurgitation; TR, tricuspid regurgitation; LVEF, left ventricular ejection fraction.

## Results

From the full pool of TTEs meeting all inclusion and exclusion criteria, a randomized and severity-enriched sample of 1541 studies was selected for external testing. Among these, the model returned predictions for 280 of 776 MR studies (36%) and 298 of 765 TR studies (39%), resulting in a final analysis cohort of 578 studies (38% of the sampled set). Studies that did not yield predictions were excluded from performance evaluation. Accordingly, model performance was assessed in 280 MR cases and 298 TR cases, corresponding to the subset of TTEs that successfully passed the internal view classification and quality control filters.

The median age in both cohorts was 76 years, and the population was predominantly non-Hispanic white (>95%). Male sex was recorded in 45% of MR patients and 51% of TR patients. Left ventricular ejection fraction was preserved across groups, with a median [IQR] of 57% [51–63] in the MR cohort and 56% [48–62] in the TR cohort.

Cardiovascular conditions were common. Arrhythmias were reported in 72% of MR patients and 79% of those with TR, while coronary artery disease was present in 19% and 24%, respectively. Hypertension, as a major cardiovascular risk factor, was observed in 59% of MR patients and 65% of TR patients. Other comorbidities included chronic pulmonary disease (40% in MR, 39% in TR) and renal disease (39% and 45%, respectively). Additional risk factors such as cerebrovascular disease, peripheral vascular disease, diabetes, and dementia were also represented.

Ground truth severity labels were distributed across four categories: in the MR cohort, 29% of studies were classified as normal, 43% as mild, 17% as moderate, and 11% as severe. In the TR cohort, 26% were graded as normal, 40% as mild, 21% as moderate, and 13% as severe. Accordingly, 29% of MR studies and 34% of TR studies met the threshold for clinically significant regurgitation. Full baseline characteristics, including ground truth severity distribution, are summarized in *Table 1*.

**Figure 2:**
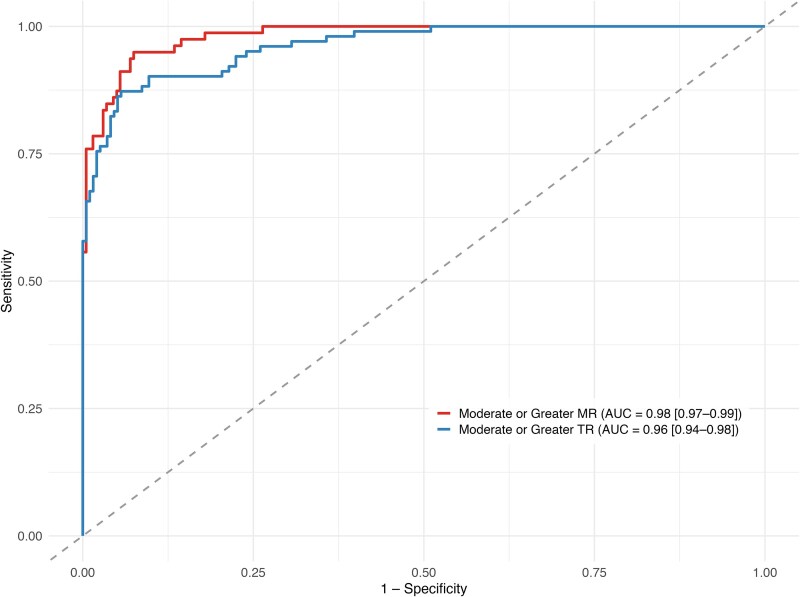
Receiver operating characteristic curves for artificial intelligence model based classification of moderate or greater mitral and tricuspid regurgitation.

The primary analysis evaluated the ability of the model to distinguish clinically significant regurgitation (defined as moderate or severe) from lesser grades (normal or mild). Performance metrics are summarized in *Table 2*, with corresponding receiver operating characteristic (ROC) curves shown in *Figure 2*. For MR, the model achieved an AUC of 0.98 (95% CI: 0.97–0.99), with sensitivity of 95%, specificity of 89%, accuracy of 91%, and F1 score of 0.85. For tricuspid regurgitation, the AUC was 0.96 (95% CI: 0.94–0.98), with sensitivity of 91%, specificity of 80%, accuracy of 84%, and F1 score of 0.79.

**Table 2:** Aisap.ai performance in classifying moderate and above mitral and tricuspid regurgitation

	AUC [95% CI]	Sensitivity	Specificity	Accuracy	F1
MR	0.98 [0.97–0.99]	0.95	0.89	0.91	0.85
TR	0.96 [0.94–0.98]	0.91	0.8	0.84	0.79

AUC, area under the curve; MR, mitral regurgitation; TR, tricuspid regurgitation.

**Figure 3:**
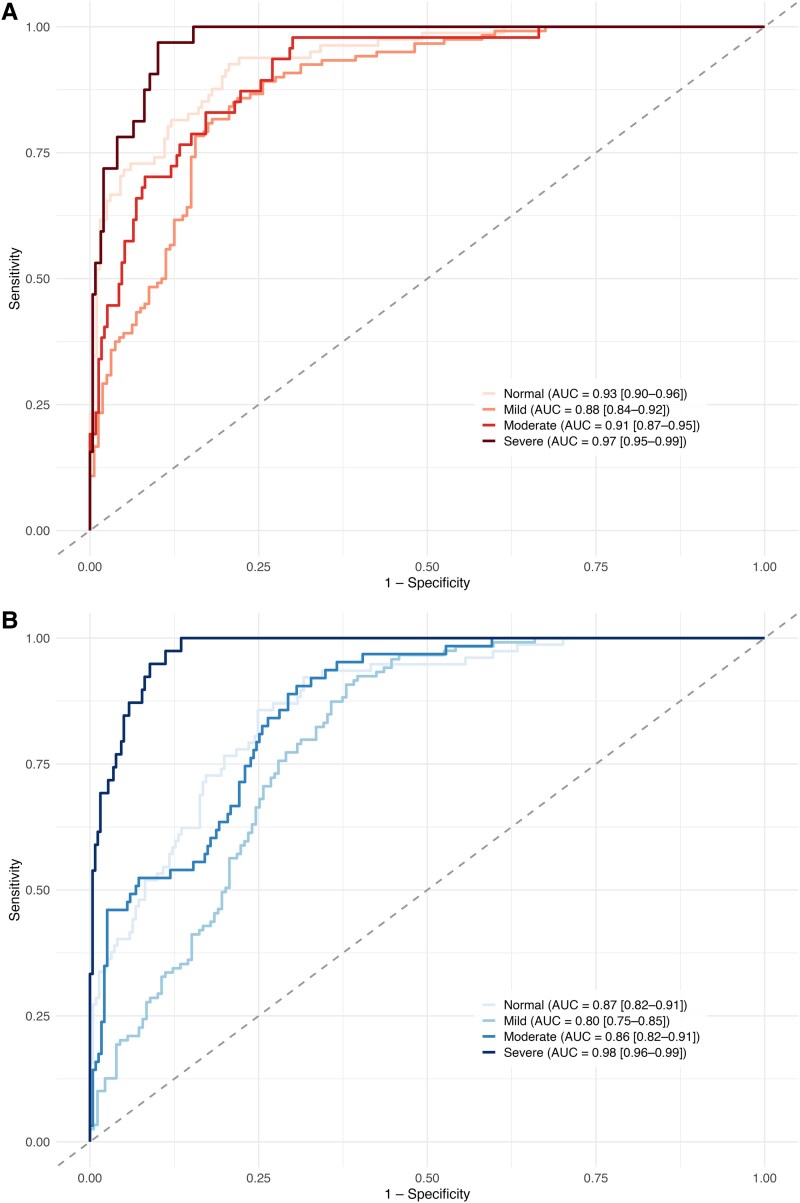
Class-specific receiver operating characteristic curves for artificial intelligence model based classification of regurgitation severity. (*A*) Mitral regurgitation. (*B*) Tricuspid regurgitation.

Class-specific performance metrics are presented in *Table 3* and illustrated in *Figure 3*. The model performed best in the severe regurgitation category for both valves, achieving AUCs of 0.97 for MR and 0.98 for TR. For severe MR, sensitivity was 78%, specificity 96%, accuracy 94%, and F1 score 0.74. For severe TR, sensitivity was 85%, specificity 92%, accuracy 91%, and F1 score 0.72. In contrast, the lowest performance was observed in the mild and moderate categories, particularly for TR. The mild TR group showed the weakest results, with a sensitivity of 37% and F1 score of 0.47. The AUC values across all severity classes ranged from 0.80 to 0.98.

**Table 3:** Class-specific performance of Aisap.ai in regurgitation severity classification

	AUC [95% CI]	Sensitivity	Specificity	Accuracy	F1
A. Mitral regurgitation					
Normal	0.93 [0.9–0.96]	0.74	0.91	0.86	0.76
Mild	0.88 [0.84–0.92]	0.68	0.85	0.78	0.73
Moderate	0.91 [0.87–0.95]	0.72	0.88	0.86	0.63
Severe	0.97 [0.95–0.99]	0.78	0.96	0.94	0.74
B. Tricuspid regurgitation					
Normal	0.87 [0.82–0.91]	0.73	0.81	0.79	0.64
Mild	0.8 [0.75–0.85]	0.37	0.87	0.67	0.47
Moderate	0.86 [0.82–0.91]	0.57	0.81	0.76	0.5
Severe	0.98 [0.96–0.99]	0.85	0.92	0.91	0.72

**Figure 4:**
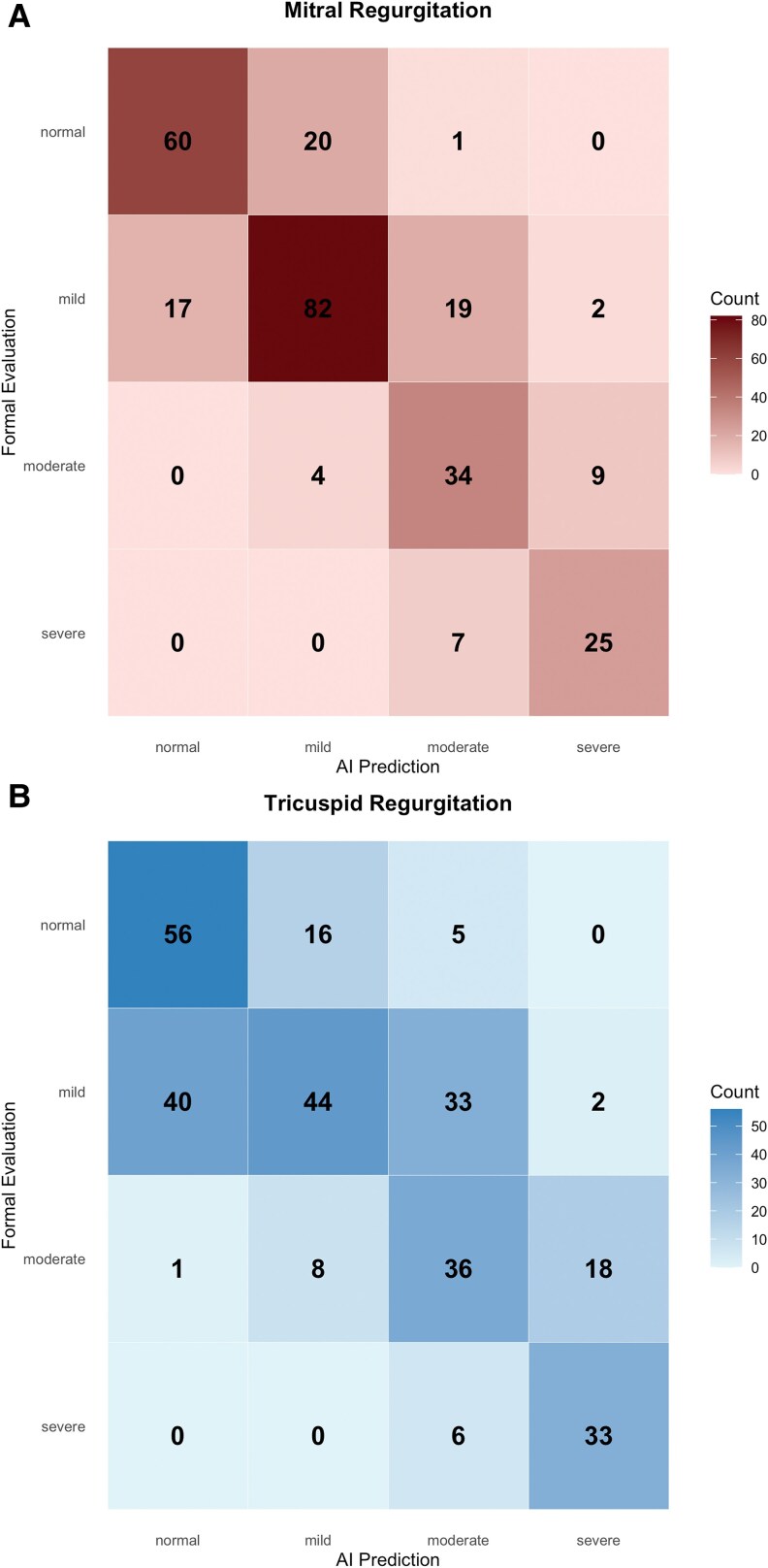
Confusion matrices for artificial intelligence model classification of regurgitation severity. (*A*) Mitral regurgitation. (*B*) Tricuspid regurgitation. Rows represent ground truth from formal echocardiographic reports. Columns show model predictions across four severity levels (normal, mild, moderate, severe).

Misclassification patterns are shown in *Figure 4*. Most errors occurred between adjacent severity categories, particularly between mild and moderate. Among all misclassified MR cases, 88.4% had ground truth labels of mild or moderate; the corresponding figure for TR was 83.7%. No severe cases were misclassified as normal or mild, and all misclassification within the severe group was limited to a one-grade shift (i.e. severe to moderate). Conversely, cases with ground truth of normal or mild were rarely predicted as severe by the model.

## Discussion

This study demonstrates that the AI algorithm can achieve high diagnostic accuracy in identifying clinically significant MR and TR using formal TTE—in cases where a prediction was successfully generated. Among this subset, the model achieved AUCs of 0.98 for MR and 0.96 for TR, with corresponding sensitivity and specificity values of 95% and 89% for MR and 91% and 80% for TR, respectively. Notably, no cases of severe regurgitation were misclassified as normal or mild, highlighting the model’s ability to differentiate clinically meaningful disease from benign findings.

Importantly, these results were obtained using a constrained input design. The model operates exclusively on A4C and PLAX views, with and without colour Doppler, and does not require user-supplied measurements or manual annotations. Predictions are generated from visual patterns learned during training, using cardiologist-assigned severity labels as the reference standard. The model’s ability to achieve strong diagnostic accuracy without reliance on full protocol imaging or manual quantification highlights the potential of streamlined AI solutions in echocardiography.

However, only approximately 35–40% of the echocardiograms processed in this external validation yielded predictions. This reflects the fact that the model was applied retrospectively in a backend fashion, without integration into the clinical workflow or access to real-time quality feedback. In its intended use, the model provides immediate feedback on input adequacy, enabling users to adjust acquisition if needed. In this study, by contrast, the algorithm was run on all eligible exams, and only those that produced a prediction were included in the analysis; studies without outputs were excluded from performance evaluation.

We hypothesize that view classification and input eligibility criteria played a central role in limiting prediction yield. Acquisition practices varied across sites due to local imaging conventions. For example, some A4C views were acquired in a ‘top-down’ orientation (atria at the top), whereas the model was trained predominantly on ‘bottom-up’ views. In PLAX views, colour Doppler was sometimes applied across both mitral and aortic valves rather than individually. These clinically acceptable variations may have fallen outside the model’s training distribution, reducing the likelihood of successful view recognition. Additional differences in frame rate, image resolution, or Doppler settings may have further limited input eligibility.

These findings highlight a broader insight: diagnostic AI tools are not plug-and-play. Model performance is tightly coupled to acquisition conditions, and integration with data capture processes is essential for reliable deployment. The ability to generalize across diverse practice settings may depend not only on algorithm architecture but also on the breadth of training data and the alignment of imaging protocols. Future development should aim to expand the model’s tolerance for acquisition heterogeneity, potentially by incorporating more diverse datasets, including those with variable orientations. Newer versions of the model are already being trained to support a wider range of inputs and are expected to improve prediction yield.

Despite these limitations, the current results demonstrate that, within eligible cases, the model delivers robust diagnostic performance. The external validation was conducted across a heterogeneous clinical landscape, capturing variability at both the population and healthcare system levels. The algorithm, originally trained on echocardiograms from an Israeli cohort, was tested on a demographically distinct US population, enabling evaluation across geographic and biological contexts. The high concordance with internal validation results^28^ suggests that, when inputs meet the required conditions, performance is preserved across clinical environments.

Beyond model-specific validation, our findings also align with prior AI-based studies that have assessed MR and TR using TTE.^33–35^ Although these models varied in architecture, inputs, and labelling strategies, they converged on the importance of integrating multiple views and Doppler data to optimize diagnostic accuracy. Notably, to the best of our knowledge, previously published models have remained research prototypes, whereas Aisap.ai is commercially available and FDA-cleared, supporting its clinical readiness and real-world applicability.

These findings illustrate the model’s potential for deployment in frontline care environments, where its ability to achieve high diagnostic performance using only basic echocardiographic views—without manual measurements—positions it as a practical and scalable solution for non-specialist settings. As POCUS devices become increasingly accessible, we envision a workflow in which primary care physicians can leverage AI-assisted image acquisition and interpretation to guide clinical decision-making. In scenarios where physical examination findings suggest possible valvular pathology, or where unexplained symptoms raise clinical concern, physicians could acquire two standard views and receive immediate automated feedback. This approach may help identify patients who would benefit from formal cardiology referral or comprehensive imaging, thereby expanding access to timely evaluation and improving care pathways for valvular heart disease.

While these primary care triage applications represent the intended use case, the algorithm may also hold value in additional clinical scenarios. One potential application is in training environments, where the model could serve as a supportive tool for early-career echocardiographers or sonographers by providing real-time feedback and an independent severity assessment during the learning curve. On an organizational level, it could theoretically be deployed in a retrospective backend fashion—similar to the approach used in this study—to scan large volumes of echocardiographic data. In such settings, automated flagging of cases with discrepant findings between the model and the original clinical report could highlight studies warranting secondary review. However, the clinical utility and operational feasibility of such use would require further validation, including an analysis of false-positive rates, resource implications, and downstream effects on workflow and diagnostic accuracy.

Several limitations pertain to the design and execution of this external validation study. First, the cohort was relatively small and deliberately balanced across severity grades, which does not reflect the true prevalence of atrioventricular regurgitation in clinical practice. In real-world populations, moderate and severe MR or TR are far less common, and this enrichment strategy—while necessary to evaluate performance across severity levels—limits the generalizability of the findings and precludes meaningful interpretation of predictive values such as positive predictive value and negative predictive value. Second, as discussed above, the retrospective design and lack of integration with acquisition workflows restricted prediction yield, and the population that received predictions may not be representative of the broader cohort. Third, our inclusion and exclusion criteria further restrict generalizability. The study was limited to TTE without contrast or strain imaging and included only patients with native valves. These choices exclude key populations such as paediatric patients, those with congenital heart disease, prosthetic valves, or mechanical circulatory support, limiting the scope of applicability.

Finally, the model does not incorporate guideline-based quantitative measurements, which may limit its utility in borderline or serial cases. While it provides categorical predictions based on learned visual patterns, it does not offer measurement-based outputs that some clinicians may prefer for transparency or tracking disease progression. In its current form, the algorithm should be viewed as a supportive tool to aid early triage, not a replacement for comprehensive echocardiographic assessment. Specifically, decisions regarding therapeutic interventions, including surgical or transcatheter procedures, must continue to be based on formal echocardiographic studies performed and interpreted within the broader clinical context.

True clinical utility will depend not only on prospective evaluation but also on thoughtful integration into real-world workflows. Embedding the model into routine care—particularly in primary care, emergency, or low-resource settings—will allow for assessment of not only diagnostic performance but also operational impact. In particular, the envisioned use of the model alongside POCUS warrants prospective trials to determine feasibility, usability, and effect on timely identification of valvular disease. Future studies should explore these implementation pathways and assess whether AI-assisted interpretation can enhance efficiency, reduce diagnostic delays, and improve patient outcomes in settings where access to expert imaging is limited.

## Data Availability

All requests for raw and analysed data and related materials will be reviewed by the Mayo Clinic’s Legal Department and Mayo Clinic Ventures to verify whether the request is subject to any intellectual property or confidentiality obligations. Requests for patient-related data not included in the paper will not be considered. Any data and materials that can be shared will be released via a Material Transfer Agreement. The code underlying the AI software is proprietary intellectual property owned by the sponsor (Aisap.ai) and is not publicly available. However, access to the software for purposes of result reproduction may be requested directly from the sponsor.
